# ﻿Two new species and a new record of the ant genus *Meranoplus* Smith, 1853 (Hymenoptera, Formicidae, Myrmicinae) from Thailand

**DOI:** 10.3897/zookeys.1210.125990

**Published:** 2024-08-26

**Authors:** Kuntima Yodprasit, Nopparat Buddhakala, Wattanachai Tasen, Weeyawat Jaitrong

**Affiliations:** 1 Department of Forest Biology, Faculty of Forestry, Kasetsart University, Bangkok, 10900, Thailand Kasetsart University Bangkok Thailand; 2 Division of Biology, Faculty of Science and Technology, Rajamangala University of Technology Thanyaburi, Pathum Thani, 12120 Thailand Rajamangala University of Technology Thanyaburi Pathum Thani Thailand; 3 Office of Natural Science Research, National Science Museum, 39 Moo 3, Khlong 5, Khlong Luang, Pathum Thani, 12120, Thailand National Science Museum Pathum Thani Thailand

**Keywords:** Biodiversity, distribution, Oriental and Indo-Australian regions, taxonomy

## Abstract

*Meranoplus* Smith, 1853 is distributed in the Old World tropics, from Africa, Asia, New Guinea to Australia. There are four species *Meranoplusbicolor* (Guérin-Méneville, 1844), *M.castaneus* Smith, 1857, *M.laeviventris* Emery, 1889, and *M.mucronatus* Smith, 1857 previously recorded from Thailand. In the present paper, two new species of the genus, *M.siamensis* Yodprasit & Jaitrong, **sp. nov.** and *M.tanomtongi* Yodprasit & Jaitrong, **sp. nov.**, are described based on the worker caste. Additionally, *M.malaysianus* Schödl, 1998 is recorded for the first time for Thailand. A key to the Oriental and Indo-Australian species, based on the worker caste, is provided. The new species and the new record were found to nest in soil.

## ﻿Introduction

*Meranoplus* Smith, 1853 is distributed in the Old World tropics from Africa, Asia, and New Guinea to Australia (Bolton 2024). Members of this genus nest in the soil, in rotten wood, or under stones (Bolton 1981; Jaitrong et al. 2020) and are known to be active both day and night (Gross et al. 1991). The genus has been revised and reviewed across its entire distribution over the past few decades (Australasia and New Guinea: Donisthorpe 1947; Andersen 2006; Taylor 2006; Schödl 2007; Africa: Bolton 1981; Madagascar: Boudinot and Fisher 2013; and Asia: Chapman and Capco 1951; Wu and Wang 1995; Schödl 1998, 1999; Terayama 2009; Guénard and Dunn 2012; Bharti and Akbar 2014; Bharti et al. 2016; Jaitrong et al. 2016; Dias et al. 2020). Currently, 91 valid species of the genus are known (Bolton 2024). Among them, 60 species have been recorded from the Australasian region, 13 from the Oriental region, eight from the Afrotropical region, and four from the Malagasy region (Boudinot and Fisher 2013; AntWiki 2024; Bolton 2024). Until now, only four species, *Meranoplusbicolor* (Guérin-Méneville, 1844), *M.castaneus* Smith, 1857, *M.laeviventris* Emery, 1889, and *M.mucronatus* Smith, 1857, were known from Thailand (Jaitrong and Nabhitabhata 2005; Jaitrong et al. 2020; Khachonpisitsak et al. 2020).

We examined *Meranoplus* specimens from Thailand and recognized seven species. *Meranoplusmalaysianus* Schödl, 1998 is newly recorded from the country, and *M.siamensis* Yodprasit & Jaitrong, sp. nov. and *M.tanomtongi* Yodprasit & Jaitrong, sp. nov. are new to science and described here based on the worker caste. A key to the Oriental and Indo-Australian species of *Meranoplus*, based on the worker caste, is presented.

## ﻿Materials and methods

This study was mainly based on the specimens deposited in the Natural History Museum of the National Science Museum, Thailand. Almost 500 specimens of the genus *Meranoplus* were examined. Specimens of the new species and the new record were compared with the images available on Antweb (2024) of holotypes and paratypes of small species distributed in Asia: *M.borneensis* Schödl, 1998; *M.loebli* Schödl, 1998; and *M.malaysianus* Schödl, 1998. A paratype of *M.malaysianus* deposited in the Seiki Yamane Collection, Kagoshima, Japan was also examined.

Most morphological observations were made with a Zeiss Discovery V12 stereoscope. Multi-focused montage images were produced using NIS-Elements-D from a series of source images taken with a Nikon Digital Sight-Ri1 camera attached to a Nikon AZ100M stereoscope. Specimens were measured for the following parts using a micrometer on a Zeiss Discovery V12 stereoscope. All measurements are given in millimeters and recorded to the second decimal place.

The abbreviations for the measurements and indices used are as follows: (edited from Bolton 1981; Hölldobler and Wilson 1990; Schödl 1998):

**HL** Head length, straight-line length of head in full-face view, measured from the mid-point of the anterior clypeal margin to the midpoint of the posterior margin. In species where one or both of these margins are concave, the measurement is taken from the mid-point of a transverse line that spans the apices of the projecting portions.

**HW** Head width, maximum width of head in full-face view, excluding the compound eyes.

**ML** Mesosomal length, the diagonal length of the mesosoma in profile from the point at which the pronotum meets the cervical shield to the posterior basal angle of the metapleuron.

**PML** Length of promesonotal shield, measured from anterior mid-point of pronotum behind collar that is the mid-point of a virtual line, where the anterior pronotal margins meet, to mid-point of behind margin of mesonotum above propodeum.

**PW** Pronotal width, measured right behind base of anterolateral pronotal projection (angle) in dorsal view.

**SL** Scape length, straight-line length of the antennal scape, excluding the basal constriction or neck.

**TL** Total length, total outstretched length of the individual, from the mandibular apex to the gastral apex.

**CI** Cephalic index, HW/HL×100.

**PMI** Pronotum index, PW/PML×100.

**SI** Scape index, SL/HW×100.

### ﻿Abbreviations of the ant collections are as follows

**AMK** Ant Museum, Faculty of Forestry, Kasetsart University, Thailand

**BMNH** The Natural History Museum, London, United Kingdom

**MCZC** Museum of Comparative Zoology, Cambridge, USA

**NHMB** Naturhistorisches Museum, Basel, Switzerland

**NHMW** Naturhistorisches Museum Wien, Vienna, Austria

**SKYC** Seiki Yamane Collection, Kagoshima, Japan

**THNHM** Natural History Museum of the National Science Museum, Thailand

Scanning electron microscope images were made at the Microscopic Center, Faculty of Science, Burapha University with a Leo 1450 VP scanning electron microscope with gold-coated specimens.

For general terminology in the worker caste of ants, see Bolton (1994) and Hölldobler and Wilson (1990). The terminology of the ant genus *Meranoplus* follows Schödl (1998).

## ﻿Taxonomy

### ﻿ Meranoplus

Taxon classificationAnimaliaHymenopteraFormicidae

Smith, 1853

E1318CC3-CE6E-5994-A993-406159238084

Meranoplus Smith, 1853: 224. Type species: Cryptocerusbicolor, by subsequent designation of Bingham (1903: 166).
Tricytarus Donisthorpe, 1947: 187. Type species: Tricytarusparviumgulatus, by original designation (junior synonym of Meranoplus by Boudinot 2014: 96).

#### Diagnosis of worker.

Bolton (1981) and Sharaf et al. (2014) defined characteristics of this genus as follows: 1) worker is distinctly monomorphic; 2) antennae 9-segmented, with three apical segments forming a club; 3) frontal scrobes distinct, deep, and long; 4) palp formula 5,3; 5) masticatory margin of mandibles with 4–5 teeth; 6) compound eyes present, usually strongly convex, located below antennal scrobes; 7) pronotal spines present, dentiform; 8) mesonotal spines present; 9) mesosomal dorsum fused to form a shield; 10) propodeal spines present; 11) petiolar spines present or absent; 12) with dense, long, erect hairs on body surface.

### ﻿ Meranoplus malaysianus

Taxon classificationAnimaliaHymenopteraFormicidae

Schödl, 1998

FC6BDA71-2E96-530F-85B9-7DC52E813717

Figs 1A–F , 7F–I

Meranoplus malaysianus Schödl, 1998: 385, figs 4, 18, 32 (workers and queen).
Meranoplus malaysianus : Pfeiffer et al. 2011: 47; Satria and Herwina 2020: 83.

#### Types.

***Holotype*** • (BMNH, CASENT0902029, images examined), West Malaysia (Malaya), Kuala Lumpur, 8 October 1973, B. Bolton. ***Paratypes***: • 10 workers and 1 queen, same locality data as holotype (BMNH, NHMW) • 3 workers, Berlese funnel, Malaysia (MALAYA), K. Lumpur, 8 October 1973, B. Bolton, *Meranoplus* sp. det. B. Bolton, 1974 (NHMB, NHMW) • 5 workers, Malaysia Neg. Sembilan Pasoh For. Res. November 1994, litt (= litter?) sample M. Brendell, K. Jackson, S. Lewis (BMNH, NHMW) • 4 workers, 2 queens, 1 male (head missing), Damm., Depok 7.1. [1 ex. 30.111.] 1923, MCZC Museum of Comparative Zoology (MCZC, NHMW) • 3 workers, F192-387, Kebun Raya, Bogor, W-Java, Indonesia, 11–31 Janaury 1992, F. Ito (NHMW).

#### Non-type material examined.

**Southern**: • 1 worker (THNHM-I-00028943, THNHM), Songkhla Province, Hat Yai District, Thung Tam Sao Subdistrict, evergreen rain forest, 27 July 2002, N. Noon-anant leg. • 1 worker (THNHM-I-00027334, THNHM), Narathiwat Province, Wang District, Lo Chood Subdistrict, 5.8455°N, 101.8756°E, 25 September 2001, R. Poonjampa leg., hand collecting • 1 worker (AMK), same locality and date, S. Hasin leg., general collection.

#### Measurements and indices.

Workers (*n* = 2): HL 0.68–0.69, HW 0.68–0.70, ML 0.65, PML 0.45–0.49, PW 0.65, SL 0.44, TL 2.79–2.84, CI 99–104, PMI 133–142, SI 63–64.

#### Diagnosis of worker.

Small species (HW 0.68–0.70 mm, TL 2.79–2.84 mm). Promesonotal shield shorter than broad and distinctly margined, broadly transparent at sides, overhanging lateral face of mesosoma; anterior corners of pronotum almost right angles; lateral margins of pronotum parallel, slightly sinuate. Petiole in profile tapered, the crest obliquely and narrowly truncate. Postpetiole nodiform, almost as long as high and roundly convex dorsal outline. Dorsa of head and promesonotal shield densely reticulate-rugulose; lateral portion of pronotum reticulate-rugulose; mesopleuron, metapleuron, and lateral faces of propodeum sparsely reticulate-rugulose, with smooth interspaces. Anterior face of petiole smooth and shiny, dorsum and lateral faces rugulose, posterior face smooth. First gastral tergite smooth, with an occasional faint shagreening around piliferous punctures.

**Figure 1.**
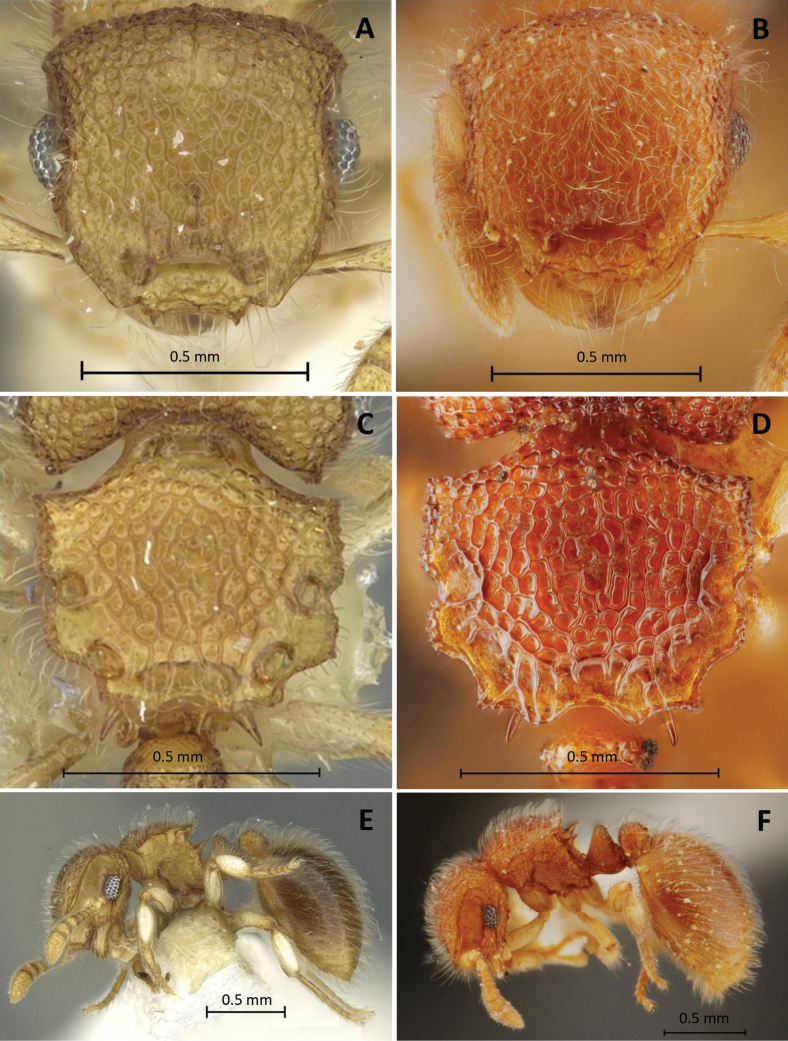
*Meranoplusmalaysianus***A, C, E** holotype (CASENT0902029) **B, D, F** non-type worker (THNHM-I-00028943) **A, B** head in full-face view **C, D** promesonotal shield in dorsal view **E, F** body in profile.

The Thai specimens agreed well with the holotype (CASENT0902029) in structure, sculpturing, and pilosity. However, body color of the specimens collected from Thailand are reddish brown, while the holotype is paler, yellow (Fig. 1). Reticulations on dorsum of head rather denser and smaller than that on the holotype (see Fig. 1A, B for comparison).

#### Distribution.

Thailand (Songkhla and Narathiwat, new record, Fig. 4), Malaysia (West Malaysia, Sabah, Sarawak), Singapore, Indonesia (Java).

#### Habitat.

Two specimens from Narathiwat Province were collected from the ground in a disturbed area near a lowland evergreen forest, near the Thai–Malay border. A specimen (THNHM-I-00028943, THNHM) from Songkhla Province was collected in a primary evergreen forest.

### ﻿ Meranoplus siamensis

Taxon classificationAnimaliaHymenopteraFormicidae

Yodprasit & Jaitrong sp. nov.

5D520310-0B00-54B7-96CE-127B6CAFD2F2

https://zoobank.org/752DDF38-72E5-4325-9B3D-4C70BEEFA1DF

**Figure 2.**
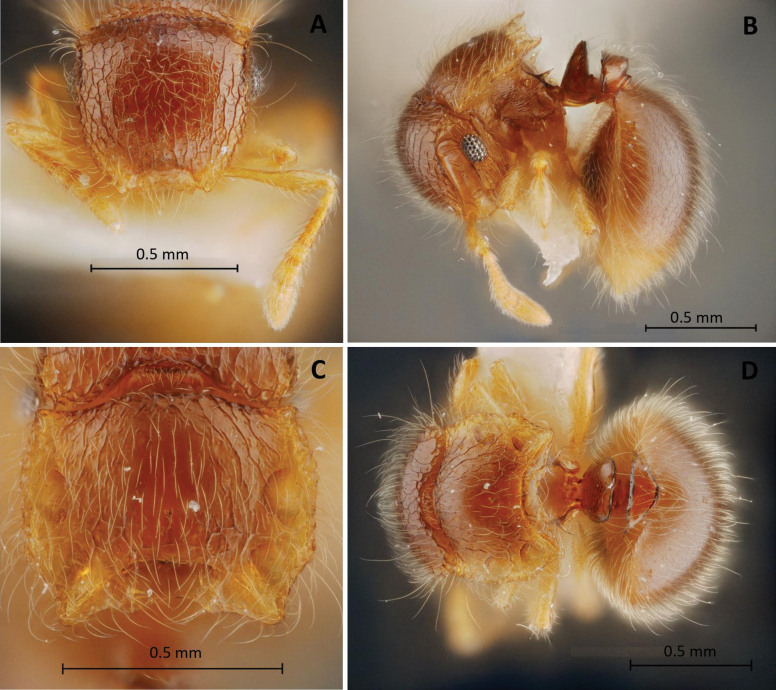
*Meranoplussiamensis* sp. nov. (holotype, THNHM-I-00027303) **A** head in full-face view **B** body in profile view **C** promesonotal shield in dorsal view **D** body in dorsal view.

Figs 2A–D , 5H–I , 6A

#### Types.

***Holotype***: • worker (THNHM-I-00027303, THNHM), eastern Thailand, Chonburi Province, Sri Racha District, Kasetsart Sriracha Campus, dry evergreen forest, 13.2837°N, 100.9238°E, 18 October 2003, W. Jaitrong leg., TH03-WJT-313.

***Paratypes***: • 6 workers (THNHM-I-00027304 to THNHM-I-0002730, THNHM-I-00027310, THNHM-I-00028942), same data as holotype • 4 workers (THNHM-I-00027309, THNHM-I-00027311 to THNHM-I-00027313) • 3 workers (THNHM-I-00027314), same locality as holotype, but 19.IV.2003, A. Suwanasri leg., AS190403-01. The paratypes are deposited in THNHM.

#### Non-type material examined.

**Central**: • 1 worker (THNHM-I-00028919, THNHM), central Thailand, Uthai Thani Province, Ban Rai District, Kaen Ma Kurd Village, dry dipterocarp forest, 15.1225°N, 99.2755°E, 1 June 2002, W. Jaitrong leg., TH02-WJT-039; 21 workers (THNHM-I-00028920 to THNHM-I-00028940, THNHM), same data locality. **Western**: • 3 workers (THNHM-I-00028941, THNHM), Kanchanaburi Province, Sai Yok District, Ban Chong Keab, dry dipterocarp forest, 25 May 2019, W. Jaitrong leg. **Northeastern**: • 7 workers (THNHM-I-00027315, THNHM), Nakhon Ratchasima Province, Wang Nam Kheao District, Sakaerat Environmental Research Station (ERS), dry dipterocarp forest, 14.5031°N, 101.9368°E, 5 June 2022, W. Jaitrong leg., TH22-WJT-264 (THNHM); **Eastern**: • 4 workers (THNHM-I-00027316, THNHM), Chachoengsao Province, Tha Takiab District, 6 April 2003, W. Jaitrong leg., WJT260403-01.

#### Measurements and indices.

Holotype worker: HL 0.63, HW 0.65, ML 0.62, PML 0.48, PW 0.63, SL 0. 0.45, TL 2.58, CI 102.38, PMI 130.21, SI 69.77. Paratype workers (*n* = 5): HL 0.60–0.65, HW 0.63–0.66, ML 0.60–0.65, PML 0.46–0.51, PW 0.62–0.65, SL 0.43–0.48, TL 2.56–2.82, CI 100–106.45, PMI 127–136.26, SI 67.72–71.97.

#### Description of worker.

***Head*** in full-face view subquadrate, slightly shorter than broad, with sides broadly convex, posterior margin distinctly convex, posterolateral corner bluntly angulate. Antennal scapes short, reaching level of posterior margin of compound eyes, apical half incrassate; antennal segment II slender, longer than each of segments III–VI, and almost as long as III+IV+V; segment VI broader than each of segments II–V. Clypeus roughly subrectangular, shorter than broad, its anterior margin feebly concave medially, while posterior clypeal margin almost straight. Mandibles subtriangular, masticatory margin with four teeth. Compound eyes large and convex when seen in full-face view, located laterally and well behind mid-length of head, with 8 or 9 ommatidia along longest axis, each facet hexagonal (Fig. 6A). Frontal lobes broad, its anterior corners right angled and lateral margin almost straight (Fig. 5H). Frontal carinae long reaching posterolateral corners of head.

***Mesosoma*** in dorsal view, promesonotal shield distinctly shorter than broad, its lateral margin convex, serrate, margined and slightly overhanging mesosoma; lateral and posterior portions of promesonotal shield with translucent fins; posterior margin of promesonotal shield sinuate and distinctly concave; anterolateral corners of promesonotal shield bluntly angulate and posterolateral corners of promesonotal shield roundly angulate; promesonotal shield with two pairs of fenestrae laterally; metanotal groove absent. Declivity of propodeum almost invisible from above, mostly overhung by posterior margin of promesonotal shield (propodeal spines are visible in profile). Mesosoma in profile subquadrate, dorsal outline weakly convex, lateral face of mesosoma relatively flat; lateral portion of pronotum subtriangular; metapleuron not clearly demarcated from mesopleuron and lateral face of propodeum. Propodeal spines long and acute, longer than wide at its base, located at middle of propodeal declivity length.

***Petiole*** in profile subtriangular. Subpetiolar process low, its ventral outline weakly convex, with small anterior denticle. **Postpetiole** in profile subquadrate, shorter than high; in dorsal view, distinctly shorter than broad, anterior margin weakly convex, posterior margin distinctly convex; dorsum of postpetiole somewhat flat, marginated with distinct ridge, posterior face convex. **Gaster** about as large as head and mesosoma combined; first gastral tergite largest, in dorsal view, its anterior margin distinctly concave.

#### Sculpture.

Mandibles striate, shiny. Antennal scapes superficially striate. Head dorsally sparsely reticulate-rugulose laterally, while median portion weakly sculptured; half posterior portion of antennal scrobes shagreened mixed with few transverse ridges. Promesonotal shield more weakly sculptured than dorsum of head, with median portion smooth, shiny, and lacking any rugae; in profile, upper one-third portion of pronotum shagreened, while lower two-third portion with sparse irregular ridges; upper one-third portion of mesopleuron shagreened, lower two-thirds longitudinal weakly striate; metapleuron, and lateral faces of propodeum somewhat smooth and shiny. Propodeum declivity shagreened. Petiole smooth and shiny, postpetiole somewhat smooth but posterior face of postpetiole scabrous. First gastral tergite superficially shagreened with smooth and shiny interspaces.

#### Pilosity and coloration.

Dorsa of head and mesosoma with dense erect hairs mixed with sparse longer hairs; antennae with dense suberect hairs; in profile, lower two-thirds of pronotum with sparse suberect hairs; lower one-third of mesopleuron and metapleuron with sparse suberect hairs; area around propodeal spiracle with sparse suberect hairs; femora and tibiae with numerous long outstanding hairs as well; petiole with weakly sparse erect hairs on its anterior face and dorsum; postpetiole with dense long erect hairs, except anterior face without hairs; gaster with dense long erect hairs. Body mainly reddish brown; mandibles, antennae, legs, and tip of gaster yellowish brown.

#### Distribution.

Thailand (Uthai Thani, Chonburi, Nakhon Ratchasima and Kanchanaburi Provinces, Fig. 4).

#### Etymology.

The specific name is after Thailand where the type locality is located; Thailand was called “Siam” in the past.

#### Habitat.

This species can be found in dry evergreen and dry dipterocarp forests. The specimens collected from northeastern Thailand (colony code TH22-WJT-264) nested in the soil. Workers moved slowly on the ground.

#### Differential diagnosis.

*Meranoplussiamensis* sp. nov. is a small species that is most similar to *Meranoplustanomtongi* sp. nov. in general appearance, having a pair of fenestrae along each lateral margin of the promesonotal shield, and having a subrectangular postpetiole when seen in profile. However, *M.siamensis* can be distinguished from *M.tanomtongi* by: 1) anterior corners of frontal lobes right angled and lateral margin almost straight (round and lateral margin weakly convex in *M.tanomtongi*, see Figs 5E, H for comparison); 2) compound eyes with 8 or 9 ommatidia along longest axis, each facet hexagon (each facet round or elliptical in *M.tanomtongi*, see Figs 5G, 6A for comparison); 3) dorsum of head weakly sculptured (dorsum of head entirely and distinctly reticulate in *M.tanomtongi*, see Fig. 5F, I for comparison); 4) dorsum of postpetiole somewhat flat, marginated with distinct ridge (shallowly concave, marginated with distinct ridge in *M.tanomtongi*); 5) entire head with dense short hairs mixed with sparse longer hairs (hairs along head margin clearly longer than hairs on middle of head in *M.tanomtongi*, see Fig. 5F, I for comparison).

The type series of *M.siamensis* sp. nov. is very similar to the non-type specimens from Central Thailand (TH02-WJT-039). However, the two colonies have some variations: 1) compound eyes with 9 ommatidia along longest axis in the type series (8 ommatidia in colony no. TH02-WJT-039); 2) promesonotal shield shorter than broad in the type series (almost as long as broad in colony no. TH02-WJT-039); 3) posterior half of head with sparse and weak reticulation in the type series (dense distinct reticulations in colony no. TH02-WJT-039); 4) propodeal declivity somewhat shagreened in the type series (smooth and shiny in colony no. TH02-WJT-039); 5) first gastral tergite superficially shagreened with smooth and shiny interspaces in the type series (distinctly shagreened in colony no. TH02-WJT-039). These characters are not clear enough to distinguish the two populations.

### ﻿ Meranoplus tanomtongi

Taxon classificationAnimaliaHymenopteraFormicidae

Yodprasit & Jaitrong sp. nov.

59ACA3DE-7390-561C-8381-8E647BA97F47

https://zoobank.org/7D7848E3-8BA7-49E5-AC8A-3A09BCE7A96B

**Figure 3.**
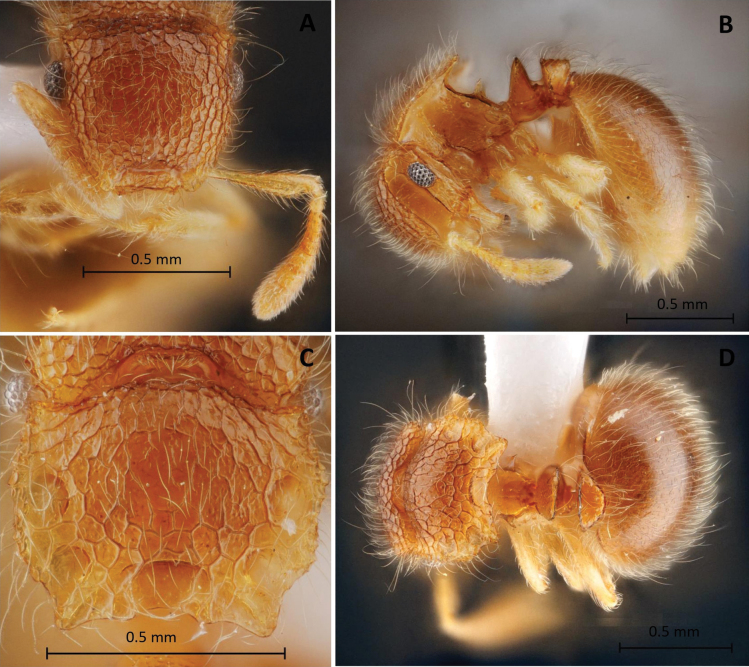
*Meranoplustanomtongi* sp. nov. (holotype, THNHM-I-00028903) **A** head in full-face view **B** body in profile view **C** promesonotal shield in dorsal view **D** body in dorsal view.

Figs 3A–D , 5A, B, E–G

#### Types.

***Holotype***: • worker (THNHM-I-00028903, THNHM), northeastern Thailand, Kalasin Province, Kuchinarai District, Nong Hang Subdistrict, dry dipterocarp forest, 16.5559°N, 104.1089°E, 10 December 2007, W. Jaitrong leg., TH07-WJT-1010, honey baiting trap. ***Paratypes***: • 10 workers (THNHM-I-00028904 to THNHM-I-00028913), same data as holotype. The paratypes are deposited in THNHM.

#### Non-type material examined.

**Laos. Central**: • 4 workers (THNHM-I-00028914, THNHM), Vientiance Province, Pak Ngum District, Ban Pha Dang, dry evergreen forest, 18.2716°N, 102.9639°E, 12 June 2010, W. Jaitrong leg., WJT10-LAO111 • 1 worker (THNHM-I-00028915, THNHM), same locality and date, Sk. Yamane leg., LA10-SKY-096, sandy soil. **Thailand. Northeastern**: • 1 worker (THNHM-I-00028916, THNHM), Mukdahan Province, Kham Cha-e District, Kheang Chang Niam Village, mixed deciduous forest, 16.5698°N, 104.2703°E, 8 June 2007, unknown collector • 1 worker (THNHM-I-00028917, THNHM), same locality, 4 August 2007, P. Kosonpanyapiwat leg. • 4 workers (THNHM-I-00028918, THNHM), same locality and collector, 2 September 2007.

#### Measurements and indices.

Holotype worker: HL 0.63, HW 0.65, ML 0.65, PML 0.51, PW 0.65, SL 0.45, TL 2.72, CI 103, PMI 129, SI 70. Paratype workers (*n* = 5): HL 0.62–0.65, HW 0.61–0.65, ML 0.62–0.68, PML 0.48–0.52, PW 0.61–0.70, SL 0.45–0.46, TL 2.62– 2.85, CI 98–104, PMI 122–140, SI 68–74.

#### Description of worker.

***Head*** in full-face view subquadrate, almost as long as broad, with sides weakly convex, posterior margin weakly convex, posterolateral corners bluntly angulate. Antennal scapes short, only reaching level of posterior margin of compound eyes, apical half incrassate; antennal segment II slender, longer than each of segments III–VI, and almost as long as III+IV+V; segment VI broader than each of segments II–V. Clypeus roughly subrectangular, shorter than broad, its anterior margin feebly concave, while posterior clypeal margin almost straight. Mandibles subtriangular, masticatory margin with four teeth. Compound eyes large, strongly convex in full-face view, located laterally behind mid-length of head, with eight ommatidia along longest axis, each facet round or elliptical (Fig. 5G). Frontal lobes broad, its anterior corners round, its lateral margin weakly convex (Fig. 5E). Frontal carinae long, reaching posterolateral corners of head.

***Mesosoma*** in dorsal view promesonotal shield distinctly shorter than broad, laterally convex, sinuate, margined and slightly overhanging mesosoma; lateral and posterior portions of promesonotal shield with translucent fins; posterior margin of promesonotal shield sinuate and distinctly concave; anterior corners of pronotum and posterior corners of mesonotum bluntly angulate; promesonotal shield with two pairs of fenestrae laterally; metanotal groove absent. Declivity of propodeum almost invisible from above, overhung by posterior margin of promesonotal shield (propodeal spines are visible in profile). Mesosoma in profile subquadrate, weakly convex dorsal outline, lateral face of mesosoma flat; lateral face of pronotum subtriangular; metapleuron not clearly demarcated from mesopleuron and lateral face of propodeum. Propodeal spines long and acute, located at middle of propodeal length, in profile.

***Petiole*** in profile subtriangular, both anterior and posterior faces weakly convex; when viewed from behind, dorsal margin transverse and smoothly convex. Subpetiolar process low, its ventral outline weakly convex, with small anterior denticle. **Postpetiole** in profile subquadrate, shorter than high; in dorsal view, distinctly shorter than broad, anterior margin almost straight, while posterior margin distinctly convex; dorsum of postpetiole shallowly concave marginated with sinuate ridge, posterior face convex. **Gaster** larger than head and mesosoma combined; first gastral tergite largest, in dorsal view, its anterior margin distinctly concave.

#### Sculpture.

Mandibles striate but shiny. Antennal scapes superficially striate. Dorsum of head in full-face view entirely reticulate; posterior half of antennal scrobes shagreened mixed with a few transverse ridges. Dorsum of promesonotal shield distinctly reticulate but median region with weaker reticulation than elsewhere; in profile, upper half portion of lateral faces of pronotum shagreened, while lower half portion with sparse irregular ridges; upper one-third portion of mesopleuron shagreened, lower two-third portion weakly longitudinally striate; metapleuron and lateral face of propodeum smooth and shiny. Propodeum declivity superficially shagreened. Petiole smooth and shiny. Postpetiole somewhat smooth but upper portion of posterior face with wrinkles. First gastral tergite superficially shagreened with smooth and shiny interspaces.

#### Pilosity and coloration.

Dorsum of head with dense erect hairs (usually a closed cell with a hair), hairs along head margin clearly longer than hairs on middle of head; antennae with dense, suberect hairs; promesonotal shield with dense, erect hairs; legs with dense suberect hairs; in profile, lower two-thirds of pronotum with sparse suberect hairs; lower one-third of mesopleuron and metapleuron with sparse suberect hairs; area around propodeal spiracle with sparse, suberect hairs; femora and tibiae with numerous long, outstanding hairs as well; petiole with sparse, erect hairs on its dorsum; postpetiole with dense, long, erect hairs, except anterior face without hairs; femora and tibiae with numerous long, outstanding hairs as well; gaster with dense, long, erect hairs. Dorsum of body (head, mesosoma, and gaster) and waist yellowish brown; mandibles, antennae, legs, and tip of gaster yellow.

#### Distribution.

**Figure 4.**
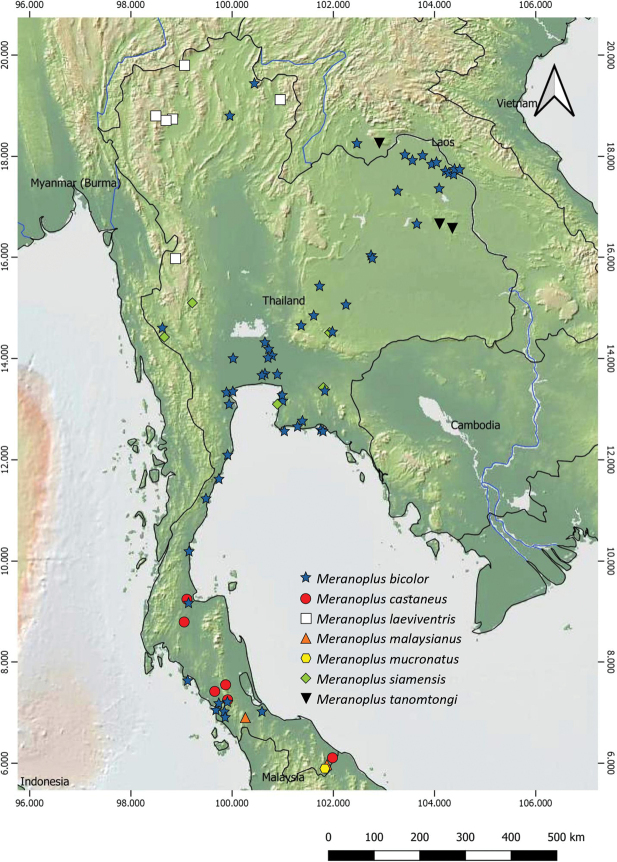
Distribution of *Meranoplus* Thai species in Thailand.

Laos (Vientiane Province), Thailand (Kalasin and Mukdahan Provinces, Fig. 4).

#### Etymology.

The specific name is dedicated to Professor Alongklod Tanomtong of Khon Kaen University, who is an excellent specialist in biological sciences in Thailand, who helped and inspired many young biologists.

#### Habitat.

This species can be found in lowland primary forest (300–600 m a.s.l.). The type series was collected from a dry dipterocarp forest. Lao specimens (colony code WJT10-LAO111) were collected from a dry evergreen forest. Specimens from Mukdahan Province, northeastern Thailand were collected in a mixed deciduous forest.

**Figure 5.**
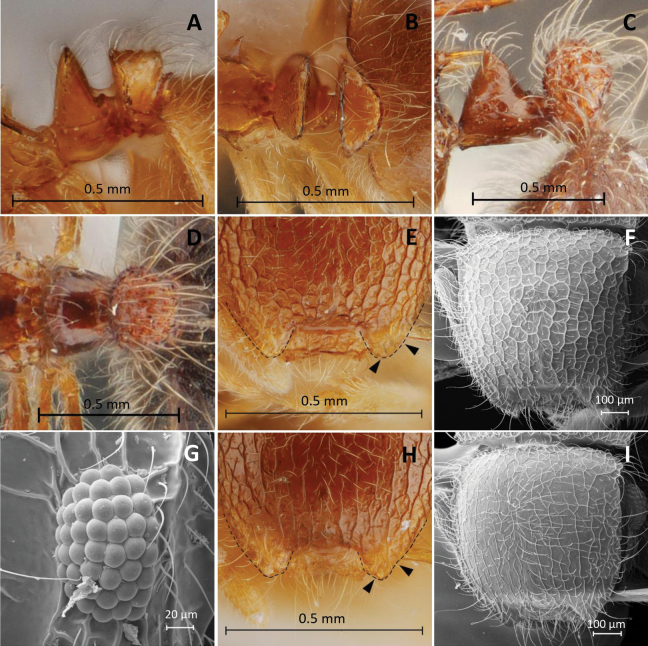
Characters used in key **A, B, E***Meranoplustanomtongi* (holotype, THNHM-I00028903) **F, G***M.tanomtongi* (non-type worker, THNHM-I-00028904) **C, D***M.bicolor* (non-type, THNHM-I-00027270) **H***M.siamensis* (holotype, THNHM-I-00027303) **I***M.siamensis* (non-type worker, THNHM-I-00028942) **A, C** petiole and postpetiole in profile **B, D** petiole and postpetiole in dorsal view **E, H** anterior corners of frontal lobes **F, I** head in full-face view **G** eye.

#### Differential diagnosis.

*Meranoplustanomtongi* sp. nov. is a small species that is most similar to *M.siamensis* sp. nov., see differential diagnosis under *M.siamensis.* This species is also similar in general appearance to *M.malaysianus* and *M.borneensis* from Sundaland, in having two pairs of fenestrae along each lateral margin of the promesonotal shield and having a concave anterior margin of first gastral tergite. However, *M.tanomtongi* can be distinguished from *M.malaysianus* and *M.borneensis* by 1) anterior corners of frontal lobes round and lateral margin weakly convex (right angled and lateral margin almost straight in *M.malaysianus* and *M.borneensis*); 2) petiole in profile subquadrate, almost flat dorsally (round, usually convex dorsal outline in *M.malaysianus* and *M.borneensis*); 3) entire lateral margin of the promesonotal shield is serrate and convex (parallel sides in *M.malaysianus* and *M.borneensis*); 4) in profile, the tip of petiole acute (truncate in *M.malaysianus* and *M.borneensis*); 5) head in full-face view entirely reticulate (densely reticulate-rugulose in *M.malaysianus* and *M.borneensis*); 6) the petiole and postpetiole are smooth and shiny (sculptured in *M.malaysianus* and *M.borneensis*).

**Figure 6.**
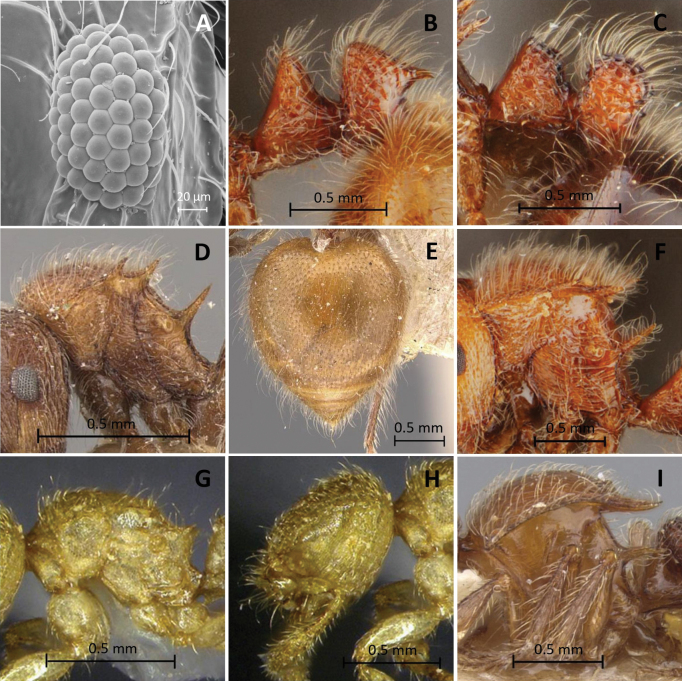
Characters used in key **A***Meranoplussiamensis* (non-type worker, THNHM-I00028942) **B, F***M.castaneus* (non-type, THNHM-I-00027323) **C***M.laeviventris* (non-type, THNHM-I-00027329) **D, E***M.bellii* (lectotype, CASENT0908933) **G, H***M.dlusskyi* (in Zryanin 2015) **I***M.levis* (holotype, CASENT0902025) **A** eye **B, C** petiole and postpetiole in profile **D, F, G, I** mesosoma in profile **E** gaster in dorsal view **H** head in profile.

### ﻿Key to the Oriental and Indo-Australian species based on worker caste (modified from Schödl 1998)

**Table:** 

1	Postpetiole in profile subquadrate (Fig. 5A); in dorsal view, postpetiole marginated with distinct ridge (Fig. 5B)	**2**
–	Postpetiole in profile nodiform and convex dorsal outline (Figs 5C, 6B, C, 7I); in dorsal view, postpetiole not marginated with distinct ridge (Fig. 5D)	**3**
2	In full-face view, anterior corners of frontal lobes round and lateral margin weakly convex (Fig. 5E); dorsum of head entirely reticulate (Fig. 5F); compound eyes with 8 ommatidia along longest axis, each facet round or elliptical (Fig. 5G)	***M.tanomtongi* Yodprasit & Jaitrong, sp. nov.**
–	In full-face view, anterior corners of frontal lobes right angled and lateral margin almost straight (Fig. 5H); median of head with weak reticulation (Fig. 5I); compound eyes with 8 or 9 ommatidia along longest axis, each facet hexagonal (Fig. 6A)	***M.siamensis* Yodprasit & Jaitrong, sp. nov.**
3	Petiolar crest distinctly bidentate; postpetiole with an acute, posteriorly directed short spine (Fig. 6B)	**4**
–	Petiolar crest never bidentate; postpetiole without an acute, posteriorly directed short spine (Figs 6C, 7I, 8E)	**5**
4	Propodeum never overhung by posterior mesonotal margin (Fig. 6D); dorsal surface of first gastral tergite smooth and brilliant except for shagreened piliferous punctures (Fig. 6E)	***M.bellii* Forel, 1902**
–	Propodeum slightly overhung by posterior translucent margin of promesonotal shield (Fig. 6F); dorsal surface of first gastral tergite dull, entirely shagreened	***M.castaneus* Smith, 1857**
5	Propodeum never overhung by the posterior mesonotal margin (Fig. 6G); compound eyes strongly reduced, consisting of 1–2 ommatidia (compound eyes completely absent in some specimens) (Fig. 6H)	***M.dlusskyi* Zryanin, 2015**
–	Propodeum overhung by the posterior mesonotal margin (Fig. 6I); compound eyes large and clearly visible, ommatidia with more 2 ommatidia (Fig. 7A)	**6**
6	Mandibles with five teeth; dorsal surface of head and promesonotum smooth, additionally distinctly carinulate (Fig. 7B)	***M.levis* Donisthorpe, 1942**
–	Mandibles with four teeth; dorsal surfaces of head and promesonotam with reticulations (Fig. 7C)	**7**
7	Promesonotal shield armed with a very long, acute spine at each corner (Fig. 7D). Large species (HL 1.4–1.7 mm, TL 5.8–7.1 mm)	***M.mucronatus* Smith, 1857**
–	Promesonotal shield without or with different armament, never with such long spines at the corners of the promesonotal shield (Fig. 7E, F). Smaller species (HL < 1.18 mm, TL < 5 mm)	**8**
8	Promesonotal shield rectangular, lacking any armament (Fig. 7F). (TL < 3.0 mm)	**9**
–	Promesonotal shield always with conspicuous, specific outstanding projections (Fig. 7G)	**10**
9	In profile, petiole distinctly obliquely truncate (Fig. 7H); first gastral tergite distinctly shagreened; pilosity consisting of short pubescence and longer outstanding hairs	***M.borneensis* Schödl, 1998**
–	In profile, petiolar crest only narrowly truncate (Fig. 7I); first gastral tergite either entirely smooth or occasionally with shagreening; pilosity on dorsal surfaces consisting of a pelt of equal sized, short hairs	***M.malaysianus* Schödl, 1998**
10	Promesonotum with only one pair of posteriorly directed mesonotal spines, without additional posterolateral and/or posterior paramedian mesonotal projections (Fig. 8A)	**11**
–	Promesonotum of different shape, always with additional posterolateral and/or posterior paramedian mesonotal projections (Fig. 8B)	**13**
11	Small species (HL 0.65–0.80 mm); promesonotal shield with a pair of posteriorly directed shorter, blunt or acute projections in posterior mesonotal corners (Fig. 8C); dorsal surfaces and appendages without extremely long hairs	***M.rothneyi* Forel, 1902**
–	Larger species (HL 0.79–0.96 mm); promesonotal shield with a single pair of posteriorly directed longer spines in posterior mesonotal corners (Fig. 8D); dorsal surfaces and appendages with long hairs	**12**
12	Dorsal surfaces of head and promesonotal shield rugose to rugulose-reticulate (Fig. 8A); petiole in profile ± an equilateral triangle (Fig. 5C)	***M.bicolor* (Guérin-Méneville, 1844)**
–	Dorsal surfaces of head and promesonotal shield shiny, with rugae and costulae (Fig. 8D); petiole in profile distinctly narrower (Fig. 8E)	***M.birmanus* Schödl, 1999**
13	Outline of lateral margins of promesonotum convex in dorsal view, each margin with two large translucent fenestrae; promesonotal shield conspicuously shorter than broad, foliaceous (PMI 178–191) (Fig. 8B)	***M.loebli* Schödl, 1998**
–	Outline of lateral margins of promesonotum in dorsal view not convex, with lateral constrictions; margins never provided with four translucent fenestrae of that size; promesonotal shield usually longer than broad (PMI 134–155), rectangular or narrowed towards hind margin, never foliaceous (Fig. 8F)	**14**
14	Mesosoma in dorsal view rectangular; lateral margins parallel-sided with a weak constriction at the level of lateral fenestrae (Fig. 8G)	**15**
–	Mesosoma in dorsal view not rectangular, lateral margins never parallel-sided, conspicuously narrowed towards posterior margin, with distinct lateral constrictions at the level of lateral fenestrae (Fig. 8H)	**17**
15	Posterior margin of mesonotum sinuate, with blunt rounded projections, lacking distinct spines (Fig. 8G); anterior margin of clypeal mid-portion produced into a serrate apron (Fig. 8I)	***M.biliran* Schödl, 1998**
–	Posterior margin of mesonotum with distinct acute paramedian spines (Fig. 9A); anterior margin of clypeal mid-portion produced into an entire, narrow apron (Fig. 9B)	**16**
16	First gastral tergite smooth and shining; body bright yellowish orange (Fig. 9D)	***M.periyarensis* Bharti & Akbar, 2014**
–	First gastral tergite entirely shagreened, anteriorly sometimes with a faint, minute reticulum; body dark brown (Fig. 9E)	***M.montanus* Schödl, 1998**
17	Petiole in profile distinctly truncate dorsally (Fig. 6C); promesonotal shield with one pair of translucent fenestrae (Fig. 8F). Larger species (HL 0.98–1.07 mm, TL 4.1–4.5 mm)	***M.laeviventris* Emery, 1889**
–	Petiole in profile cuneate dorsally (Fig. 9C); promesonotal shield with two pairs of translucent fenestrae (Fig. 9F). Smaller species (HL 0.7–0.83 mm, TL 3.0–3.4 mm)	**18**
18	In dorsal view, promesonotal shield with distinct spine-like posterolateral and posterior projections (Fig. 8H); anterior margin of clypeal mid-portion denticulate (Fig. 9G)	***M.boltoni* Schödl, 1998**
–	In dorsal view, promesonotal shield with spine-like projections only posteriorly (Fig. 9F); anterior margin of clypeal mid-portion produced into a narrow, medially excavated apron (Fig. 9H)	***M.nepalensis* Schödl, 1998**

**Figure 7.**
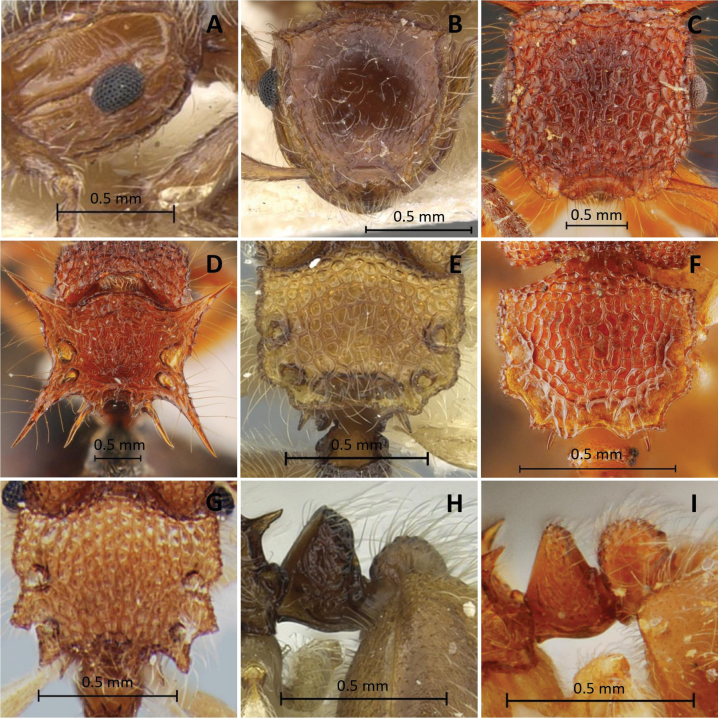
Characters used in key **A, B***Meranopluslevis* (holotype, CASENT0902025) **C, D***M.mucronatus* (non-type, THNHM-I-00027335) **E, H***M.borneensis* (paratype, CASENT0902030) **F, I***M.malaysianus* (non-type, THNHM-I-00028943) **G***M.periyarensis* (in Bharti and Akbar 2014) **A** eye **B C** head in profile **D, E, F, G** promesonotal shield in dorsal view **H, I** petiole and postpetiole in profile.

**Figure 8.**
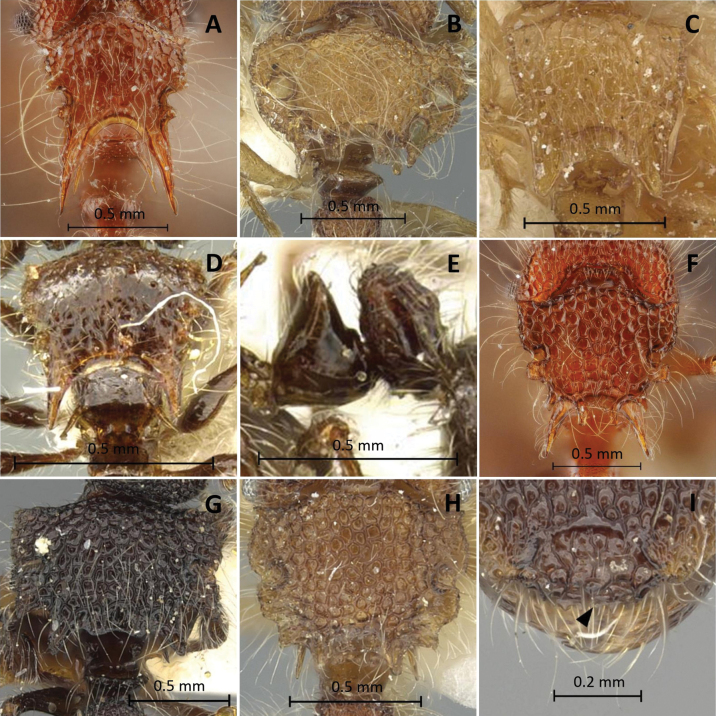
Characters used in key **A***Meranoplusbicolor* (non-type, THNHM-I-00027254) **B***M.loebli* (paratype, CASENT0902032) **C***M.rothneyi* (lectotype, CASENT0915542) **D, E***M.birmanus* (holotype, CASENT0919716) **F***M.laeviventris* (non-type, THNHM-I-00027329) **G, I***M.biliran* paratype, CASENT0902033) **H***M.boltoni* (holotype, CASENT0902031). **A–D, F–H** promesonotal shield in dorsal view **E** petiole and postpetiole in profile **I** clypeus in full-face view.

**Figure 9.**
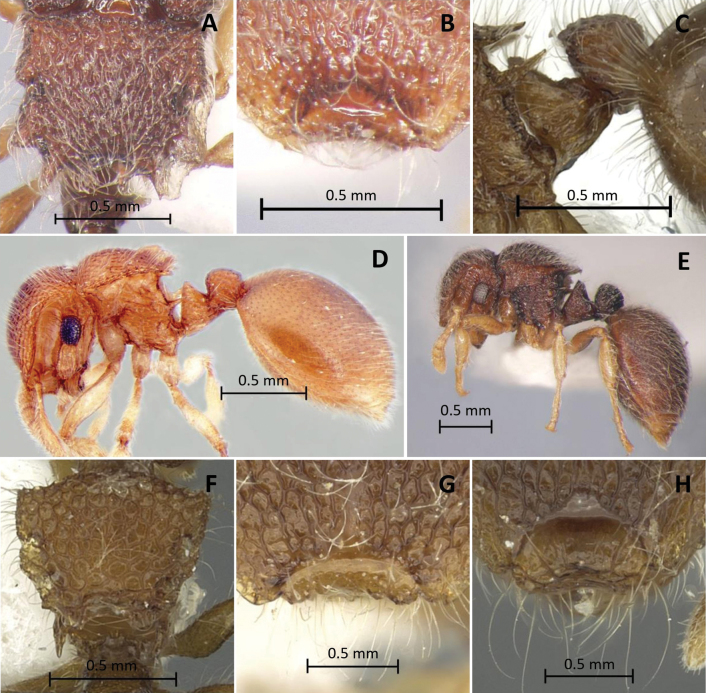
Characters used in key **A, B, E***Meranoplusmontanus* (in Bharti and Akbar 2014) **C, G***M.boltoni* (holotype, CASENT0902031) **D***M.periyarensis* (in Bharti and Akbar 2014) **F, H***M.nepalensis* (paratype, CASENT0902026) **A, F** promesonotal shield in dorsal view **B, G, H** clypeus in full-face view **C** petiole and postpetiole in profile **D, E** body in profile.

## ﻿Discussion

Until now, 19 species (including the two new species) of the genus *Meranoplus* have been known from the Oriental and Indo-Australian regions. Among them, seven species are found in Thailand (*Meranoplusbicolor*, *M.castaneus*, *M.laeviventris*, *M.malaysianus*, *M.mucronatus*, *M.tanomtongi* sp. nov., and *M.siamensis* sp. nov.). The presence of a pair of spines or teeth upon the petiolar dorsum, shape of propodeal spines, shapes of petiole and postpetiole were used by Bolton (1981) to distinguish the three species groups (*M.magrettii*, *M.nanus*, and *M.spininodis* groups) of the genus *Meranoplus* in the Ethiopian zoogeographical region. For Thai species, *M.castaneus* share a pair of spines or teeth on the petiolar dorsum with the members of *M.spininodis* group, but the other characters, such as shapes of promesonotal shield and postpetiole are different from the species group. For the moment, we do not place *M.castaneus* in the *M.spininodis* group. The other Thai species do not fit in with any species groups from Ethiopian region. Schödl (2007) separated the *M.diversus* species group from other groups in Australia by the clypeal morphology. So far, no Thai species belongs to the *M.diversus* group. Boudinot and Fisher (2013) divided the Madagascan *Meranoplus* species into two species groups (*M.mayri* and *M.nanus* groups) using sculpturing of the head and promesonotal shield, and the length of propodeal spines. The *M.nanus* species group has the subtriangular petiole and the round postpetiole, thus *M.bicolor* from Southeast Asia should belong to *M.nanus* species group. Schödl (1998) revised the oriental species of *Meranoplus* based on external morphology of the worker caste. He did not mention the species groups. We followed his morphological characters to distinguish the Thai species of the genus.

The body size, the length of posterior corners of mesonotum and propodeal spines, the shape of promesonotal shield, and the sculpturing on the first gastral tergite were used to distinguish the species of *Meranoplus* in previous papers (Bolton 1981; Schödl 1998, 2007; Boudinot and Fisher 2013). We also use these characters to separate the Thai species. The shape of frontal lobes is an important characteristic that can be used to distinguish the two new species (see couplet 2 in the key; see also Fig. 5E, H for comparison). This character was not used in the previous works. The two new species are small, and they have the subrectangular postpetiole when seen in profile and its dorsum is almost flat or shallowly concave. These characters are unique within *Meranoplus*, and thus the new species are placed in a distinct group (*Meranoplussiamensis* species group).

Members of the ant genus *Meranoplus* can be found throughout Thailand from lowland to highland (Fig. 4). *Meranopluscastaneus*, *M.malaysianus*, and *M.mucronatus* were mainly found in Sundaland (Borneo, Indonesia, and Malaysia) (Schödl 1998). Recently, only *M.castaneus* and *M.mucronatus* were recorded in Thailand. *Meranoplusmalaysianus* is recorded for the first time in the country. In Thailand, these three species are restricted to the south. The northernmost limit distribution range of *M.malaysianus* is in Songkhla Province (ca 530 km south of the Isthmus of Kra).

All species of the ant genus *Meranoplus* in Thailand nest in soil and are usually found walking on the ground except *M.castaneus*, which nests in dead branches in the canopy (ca 35 m above the ground in evergreen and swamp forests). Itino and Yamane (1995) collected *M.castaneus* in the canopy (25–35 m above ground) in mixed dipterocarp forest in Malaysia. Jantarit et al. (2009) and Watanasit et al. (2007) also found *M.castaneus* high on trees in evergreen forests. *Meranopluscastaneus* can be identified as an arboreal ant.

The two new species were found to nest in soil and walk on the ground. *Meranoplussiamensis* sp. nov. was found in the dry evergreen forest in eastern Thailand and in the dry dipterocarp forest in the western, northeastern, and central parts of the country. *Meranoplustanomtongi* sp. nov. was collected from dry dipterocarp and mixed deciduous forests in northeastern Thailand. This species was also found in a dry evergreen forest in Laos (colony No. WJT10-LAO111 and WJT10-LAO111), but the body size of Lao population is slightly larger than the type series.

## Supplementary Material

XML Treatment for Meranoplus

XML Treatment for Meranoplus malaysianus

XML Treatment for Meranoplus siamensis

XML Treatment for Meranoplus tanomtongi

## ﻿Additional information

### Author ORCIDs

Kuntima Yodprasit https://orcid.org/0009-0000-5012-0498

Nopparat Buddhakala https://orcid.org/0000-0002-8353-3851

Wattanachai Tasen https://orcid.org/0009-0004-1794-6940

Weeyawat Jaitrong https://orcid.org/0000-0003-1362-0754

### Data availability

All of the data that support the findings of this study are available in the main text.
